# A phenomics approach reveals interspecific differences in integrated developmental responses to chronic elevated temperatures

**DOI:** 10.1242/jeb.245612

**Published:** 2023-06-29

**Authors:** Jamie C. S. McCoy, John I. Spicer, Simon D. Rundle, Oliver Tills

**Affiliations:** Marine Biology and Ecology Research Centre, University of Plymouth, Drake Circus, Plymouth PL4 8AA, UK

**Keywords:** Development, Thermal biology, Embryo, Comparative physiology, Phenotyping, Gastropods

## Abstract

Phenomics, high-dimensional organismal phenotyping, is advanced as a solution to quantifying complex developmental responses to elevated temperatures. ‘Energy proxy traits’ (EPTs) measure the phenotype as a spectrum of energy values across different temporal frequencies from pixel value fluctuations of video. Although they have proven effective in measuring the biology of complex and dynamic developing organisms, their utility in assessing environmental sensitivity of different species is untested. Using EPTs, we assess the relative thermal sensitivities of embryos of three species of freshwater snail with marked differences in their developmental event timings. Embryos of *Lymnaea stagnalis*, *Radix balthica* and *Physella acuta* were videoed hourly for the duration of their embryonic development at two temperatures: 20°C and 25°C. The video was used to calculate EPTs for the duration of their embryonic development, and during discrete physiological windows in development. Changes in energy spectra during development identified marked differences in thermal sensitivities between species, and suggest a relatively heightened sensitivity of gross rates of embryonic physiology and behaviour in embryos of *R. balthica*, developmental-window-specific thermal responses that reflect ontogenetic differences in observable physiologies, and temperature-induced changes in physiological event timing. EPTs enabled comparison of high-dimensional spectral phenotypes, providing a unique capability for assessing sensitivity continuously in developing individuals. Such integrative and scalable phenotyping is a prerequisite for improved understanding of the sensitivity of early life stages of different species.

## Teaser

**Summary:** Complex, ontogenetic differences in temperature-induced responses of the phenome of encapsulated gastropod embryos are underpinned by altered physiology and behaviour, and thermal plasticity in developmental event timings.

## INTRODUCTION

Assessing the thermal sensitivity of the phenotype during early development is central to predicting how species will respond to forecasted climate change (Burggren, 2018, 2021). Chronic elevated temperatures affect processes at every level of biological organisation (Hochachka and Somero, 2002; Iverson et al., 2020), resulting in altered absolute and relative timings of organismal development (Johnston, 1993; Gillooly et al., 2002; Gomez-Mestre and Buchholz, 2006; Klimogianni et al., 2004), and rates of numerous aspects of organismal physiology (Birchard and Reiber, 1996; Styf et al., 2013; Du and Shine, 2015), behaviour (Oppenheim and Levin, 1975; Peterson and Robichaud, 1983; Peterson et al., 2004; Du and Shine, 2015; Tills et al., 2018) and size at hatch (Pepin et al., 1997; Angilletta and Dunham, 2003; Mitz et al., 2019). Despite such broad-scale changes, current approaches to measuring the response of the phenotype typically rely on reductionist approaches involving the measurement of single or a small numbers of traits with some pre-established functional significance, or in some cases, the use of gross indicators of organismal performance (e.g. metabolic rate, developmental rate, size at hatch).

It is widely acknowledged that measurement of a small number of traits may lead to erroneous conclusions over the significance of an environmental stressor in influencing the sensitivity of a developing individual, as absence of plasticity in an observed trait may be compensated for by plasticity in another, unobserved trait (Pigliucci and Preston, 2004; Houle et al., 2010; Valladares et al., 2007; Whitman and Agrawal, 2009). Although such univariate approaches are important in providing indications of organismal performance and fitness, understanding the physiological mechanisms underlying such broad-scale organismal changes requires approaches capable of quantifying high-dimensional phenotypic change (Forsman, 2015), in a manner comparable to the global approaches used in the global molecular-omics (Todgham and Hofmann, 2009; Meyer et al., 2015; Clark et al., 2017; Collins et al., 2017). Phenomics, the high-throughput acquisition of phenotypic data at the scale of the whole organism (Houle et al., 2010), is a technology-enabled approach that overcomes these limitations, and is routinely used within experimental contexts of plant biology and their interactions with environmental change. However, its use to understand animal responses to global environmental change is still in its infancy (Lürig et al., 2021).

Phenomics has enabled the tackling of key challenges including the production of drought-resistant crops in plant sciences, and identifying disease phenotypes in biomedicine (Furbank and Tester, 2011; Großkinsky et al., 2015; Neto and Borém, 2015; Alexandrov et al., 2016; Tardieu et al., 2017; Davatzikos et al., 2018). Despite the pressing need to assess phenotypic sensitivity to global climate change, the use of phenomics in environmental physiology and developmental biology remains comparatively scarce (Tills et al., 2018, 2021, 2023). However, ‘energy proxy traits’ (EPTs) have emerged as a tractable approach to phenomics when using early life stages as objects of study (Tills et al., 2018, 2021). EPTs are a spectrum of energy within different temporal frequencies in the pixel brightness fluctuations in videos of developing embryos. Rather than identifying and then targeting specific aspects of physiology or behaviour, EPTs integrate all biological sources of fluctuations in average pixel brightness in video as a spectrum of energy across discrete temporal frequency bins. Given that EPTs measure changes in pixel values in video, rather than targeting specific aspects of organismal physiology, behaviour or morphology, they should be amenable to transferability between species with markedly different developmental itineraries, and periods of development with differences in observable phenotypes. Previous work suggested that EPTs may be related to biochemical energy turnover in developing embryos (Tills et al., 2021), and although EPTs have proven effective at characterising acute and chronic responses to environmental stress in embryos of aquatic invertebrates (Tills et al., 2018, 2021), they remain untested in their capacity for interspecific comparisons. This is a major prerequisite to establishing their utility as a comparative approach to phenomics, and in assessing species-specific thermal sensitivity.

Biological development is characterised by high degrees of functional and spatial change, necessitating a focus on small windows of development, or applying limited phenotyping approaches that are applicable to observable traits between stages of development that vary in their observable phenotypes. Furthermore, biological development involves a large number of traits, with individual trait plasticities leading to considerable complexity in considering the effects of environmental drivers such as temperature (Hochachka and Somero, 2002; Iverson et al., 2020). These factors, combined with substantial variation in the timings of physiological development between closely related species (‘heterochrony’; Smith, 2002; Bininda-Emonds et al., 2007; Smirthwaite et al., 2007; Keyte and Smith, 2014), have resulted in an obstacle to organismal development being routinely considered in assessing sensitivity to environmental drivers, despite a general acknowledgement that responses at this stage are critical (Fuiman et al., 1998). Previous solutions to these limitations have centred on the use of standardised indicators of development stages such as ontologies when comparing interspecific responses at various stages of development (Walls et al., 2019), and the use of equivalent developmental events for interspecific comparisons (e.g. Smirthwaite et al., 2007). However, these approaches rely on collapsing the high-dimensional continuum of biological development into a simplified framework, potentially reducing the power of the resulting research, and its transferability to other non-model species of interest.

Consequently, here we test the thermal sensitivity of the phenome of embryos alongside pre-established heterochronic differences in physiological event timings. Additionally, we aimed to understand how thermal responses of the phenome of embryos varied between windows of development that vary in their observable physiologies, hereafter referred to as ‘physiological windows’. To do this, we applied EPTs to test the relative thermal sensitivities of three species of freshwater pulmonate gastropod (*Lymnaea stagnalis*, *Radix balthica* and *Physella acuta*) to chronic elevated temperatures. These species can occur in highly thermally variable habitats, and although data are available on acute responses of adults (Hoefnagel and Verberk, 2017; Johansson and Laurila, 2017), data on integrated developmental responses to different chronic temperatures remain limited. These species belong to the first invertebrate clade for which heterochrony was empirically revealed, in a broad range of physiological developmental events. Evolutionary differences in the relative timings of physiological events during their development (heterochronies; Gould, 1977) included species within the family Physidae (*Physella acuta*) exhibiting a significantly earlier onset of attachment to the wall of the egg capsule and commencement of muscular crawling relative to the onset of cardiovascular function, and a number of other physiological events. Conversely, in the Lymnaeidae (*Lymnaea stagnalis* and *Radix balthica*), embryos develop cardiovascular function during a free-swimming stage prior to this attachment and onset of muscular crawling (Smirthwaite et al., 2007). Consequently, these species provide an excellent model for assessing differential thermal sensitivity in high-dimensional phenotypic space, underpinned by evolutionary divergences in development, enabled by EPTs.

## MATERIALS AND METHODS

### Embryo collection

Adult snails *Lymnaea stagnalis* (Linnaeus 1758), *Physella acuta* (Draparnaud 1805) and *Radix balthica* (Linnaeus 1758) were collected from field sites in Devon and Somerset, UK (*L. stagnalis* and *P. acuta* – Exeter Canal, 50°41′57.8″N 3°30′43.7″W, April 2021; *R. balthica* – South Drain, 51°11′23.9″N 2°52′47.9″W, April 2017), at field temperatures within the range of 14.6–16°C. Adults were returned to the laboratory within 24 h of collection in buckets containing water and pondweed from the collection site. There, they were maintained in standard laboratory conditions in rearing aquaria (volume=14 litres) containing constantly aerated artificial pond water (APW) (CaSO_4_ – 120 mg l^−1^, MgSO_4_ – 245 mg l^−1^, NaHCO_3_ – 192 mg l^−1^, KCl – 8 mg l^−1^) at 15°C under a 12 h:12 h light:dark regime. Stock populations were maintained for a minimum 2-week acclimation period to minimise confounding effects owing to the recent thermal histories of individuals (Terblanche and Chown, 2006; Calosi et al., 2010). Approximately 45 adults of each species were maintained across nine containers (three containers per species, *N*=15 per container, volume=12 litres). During this time, water was changed weekly and adults were fed spinach and lettuce *ad libitum*. Egg masses (*L. stagnalis N*=3, *R. balthica N*=6, *P. acuta ­N*=3) were removed from the walls of rearing aquaria using a thin piece of laminate plastic within 24 h of deposition. On inspection (10–40×, HM-4, Microtech, UK), those that had not developed beyond the 4-cell stage were extricated from the egg mass and removed. Eggs from each egg mass were then evenly distributed between two microtitre plates (Nunc, Microwell, 96 wells, 350 µl well^−1^), with each microtitre plate held at one of two different temperatures (20 or 25°C).

### Temperature exposure and bioimaging

An open-source autonomous video microscope (OpenVIM) (Tills et al., 2018) was used to record embryonic development from the 4-cell stage to hatching. Two microtitre plates containing embryos from each of the egg masses were placed into incubation chambers (H101-K-Frame, Okolab, Italy) of two separate imaging systems, each corresponding with a different rearing temperature (20 or 25±0.2°C). A total of 96 embryos was used for each species (*N*=48 per temperature treatment). The temperature of these incubation units was controlled by circulation of water through the chamber supplied by a temperature bath (H101-CRYO-BL, Okolab), and air was supplied using an air pump (OKO AP, Okolab). Air was pre-humidified using a humidity module to minimise evaporation in wells (Okolab), and water levels were checked every 48 h and topped up using Milli-Q water (Merck, Germany) as required.

A charged couple device digital camera (resolution: 2048×2048 pixels, Pike F421B, Allied Vision, Germany) attached to an inverted lens at 200× magnification (VH-200R, Keyence, UK) was used to acquire image sequences of individual embryos. Dark field illumination was achieved using an LED ring light placed above the incubation chamber (LDR2-42-SW2, CCS, UK). The position of the incubation chamber relative to the camera was controlled using a motorised XY stage (SCAN 130×85, Märzhäuser Wetzlar, Germany). Camera and motorised stage were controlled autonomously for the duration of the experiment using the ImageJ plugin µManager (Edelstein et al., 2010). Embryos were imaged every hour for 30 s at 30 frames s^−1^ and a resolution of 1048×1048 pixels for *R. balthica*, and at 48 frames s^−1^ and a resolution of 512×512 pixels for both *P. acuta* and *L. stagnalis*. Video for *R. balthica* was obtained from a previous study, described in Tills et al. (2018, 2021).

### Image analysis

Manual analysis of the video time series for each developing embryo (*N*=48 for each species and temperature) was carried out to ascertain the timings of a number of key physiological developmental events. These events were: (i) the onset of ciliary driven rotation; (ii) the onset of cardiovascular function (determined by first visible heart beat); (iii) attachment to the wall of the egg capsule and the transition to muscular crawling behaviour using the foot; and (iv) the onset of radula function (the last developmental stage before emergence from the egg capsule in all three species) (Smirthwaite et al., 2007). These developmental events were used to delineate major physiological windows of developing embryos.

EPTs were calculated from the individual image sequence captured at each time point for each replicate embryo using an open-source Python package Embryo Computer Vision (EmbryoCV) (Tills et al., 2018). Within each 30 s time point, mean pixel values were calculated for each frame across the entire area of a bounding box surrounding the embryo, segmented autonomously by EmbryoCV. Signal decomposition of fluctuations in these mean pixel values between frames using Welch's method was used to calculate EPTs (Welch, 1967). Temporal frequency data were binned (0.1 Hz intervals to a maximum frequency of 6 Hz), producing a total of 60 frequency bands. Data were restricted to this frequency to minimise the influence of frequencies greater than those associated with any signal observable from the embryos (J.C.S.M., personal observation). Total energy, the sum of energy in all frequency bands for each time point, was calculated to produce a proxy for gross rates of embryonic physiology and behaviour (Tills et al., 2018, 2021). To standardise rates of development and enable direct comparisons between species and temperatures, the absolute timings from the 4-cell stage to hatching were converted to relative time (0–1).

### Dimensionality reduction and statistical analyses

All data were analysed in R v4.0.3 (https://www.r-project.org/). Interspecific differences in the developmental response to chronic elevated temperatures were investigated using a repeated-measures ANOVA of time series of total energy data. *Post hoc* analyses (Tukey’s HSD) were used to test for pairwise differences between temperature treatments in total energy at each point in relative developmental time. To investigate differences in response to chronic elevated temperatures between different physiological windows in development, principal component analysis (PCA) was applied using the R function prcomp() (package stats, v4.0.3). Mean values of energy within each frequency band were calculated at four key stages of development outlined above (rotation, heart, crawling and radula) for each temperature (20°C and 25°C). PCA was applied to logged EPT data and eigenvectors were used to investigate combinatorial signals from EPTs at different temperatures and physiological windows of development.

To record how responses to chronic elevated temperatures change between different physiological windows, pairwise differences in energy within discrete temporal frequency bands between temperatures within each physiological window were analysed using a multivariate Kruskal–Wallis test. To minimise false discovery rates, a Bonferroni correction was applied (*P*=0.00083).

## RESULTS

### Interspecific differences in the developmental response to chronic elevated temperatures

**Fig. 1.**
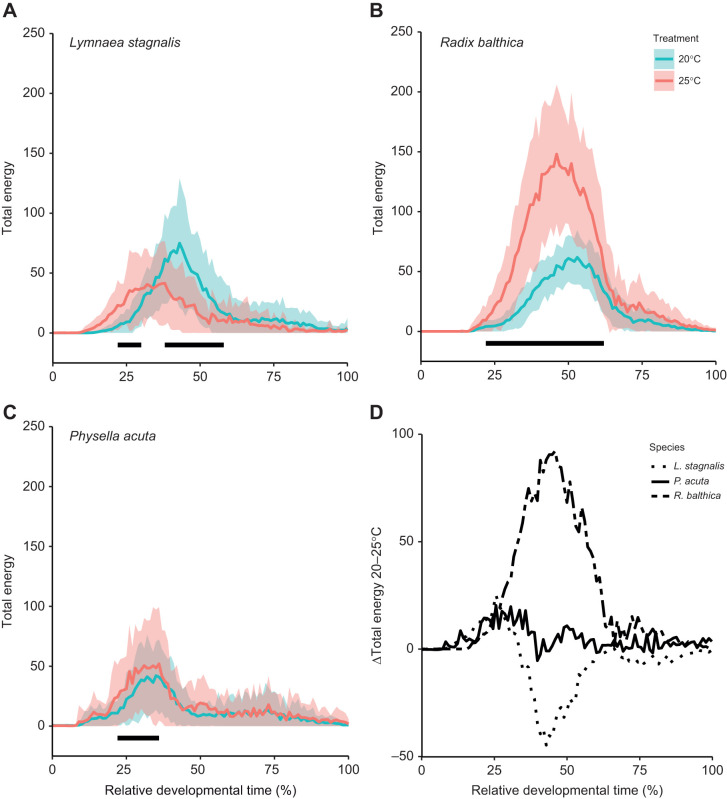
**Developmental time series of total energy (mean/1000±s.d.) in developing embryos reared at 20 and 25°C, and the associated magnitude of change between temperature treatments (mean/1000).** (A) *Lymnaea stagnalis* (20°C, *N*=32; 25°C, *N*=26), (B) *Radix balthica* (20°C, *N*=40; 25°C, *N*=37) and (C) *Physella acuta* (20°C, *N*=43; 25°C, *N*=41), and (D) change in total energy between 20 and 25°C for each species. Time is normalised (0–1) between the 4-cell stage and hatching. Black lines indicate regions of relative developmental time in which total energy is significantly different between temperature treatments (Tukey’s HSD, *P*<0.05).

Time series of all energy across the EPT spectrum (hereafter referred to as total energy) at each hourly time point revealed differences in the magnitude of response to chronic elevated temperatures between embryos of each species (Fig. 1D). Total energy (the sum of energy within all frequency bands at a particular time point) showed the greatest magnitude of change in *R. balthica*, which was significantly increased at 25°C relative to 20°C (repeated-measures ANOVA, *F*_1,99_=67.94, *P*<0.0001). Embryos at 26–62% of relative developmental time exhibited a significant increase in total energy at 25°C (Tukey’s HSD, *P*<0.0059; Fig. 1B; Table S1). Conversely, in *P. acuta*, we observed a considerably lower magnitude of change and increases in total energy at far fewer points in relative developmental time (repeated-measures ANOVA, *F*_1,99_=3.16, *P*<0.0001) (22–36% relative developmental time, Tukey’s HSD, *P*=0.034; Table S1) (Fig. 1C). However, in *L. stagnalis*, we observed both significant increases and decreases in total energy at 25°C relative to 20°C (*F*_1,99_=21.16, *P*<0.0001). At approximately 22–30% relative developmental time, we observed a decrease in total energy (Tukey’s HSD, *P*<0.042; Table S1), whereas at 38–58% relative developmental time, total energy was increased (Tukey’s HSD, *P*<0.0001; Table S1) (Fig. 1A).

Temperature-related differences in the relative timings of development were also evident from time series of total energy (Fig. 1). For *L. stagnalis*, these shifts in timing were evident as the total energy time series trend at 25°C being shifted forward in relative developmental time, relative to that at 20°C (Fig. 1A). Manual determination of the absolute timings of major developmental events used in this study showed that all were accelerated at 25°C relative to 20°C (Kruskal–Wallis, *P*<0.001; Table 1; Table S2). Temperature-induced changes in physiological event timings were also observed in *R. balthica*, and manual quantification of developmental events revealed an acceleration of the onset of muscular crawling and cardiovascular function (Kruskal–Wallis, *P*<0.001; Table S2).

**Table 1.**
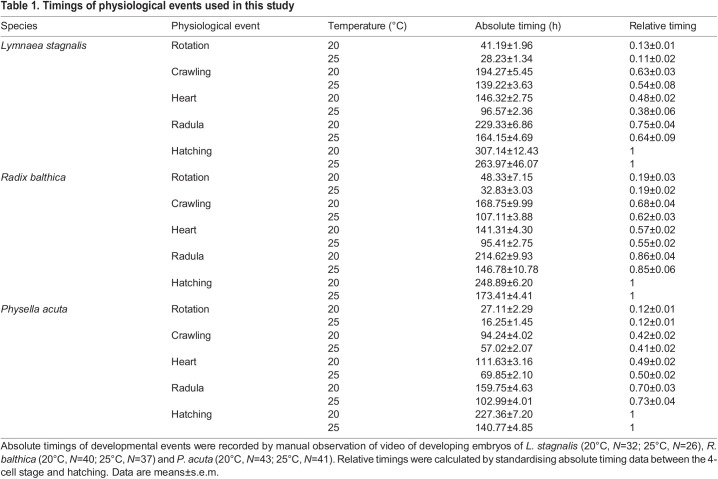
Timings of physiological events used in this study

### Differences in thermal responses between physiological windows in development

**Fig. 2.**
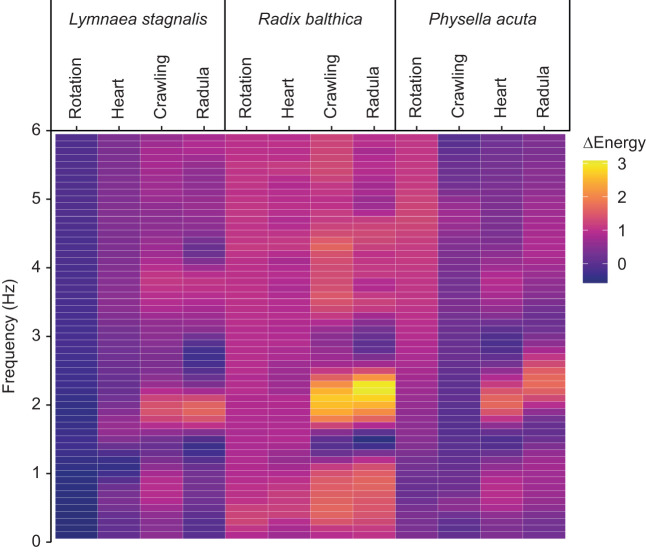
**Magnitude of change in energy across 60 temporal frequency bands at four key physiological windows in development, between embryos of *L. stagnalis*, *R. balthica* and *P. acuta* reared at 20 and 25°C.** Rotation, onset of ciliary driven rotation; Heart, onset of cardiovascular function; Crawling, attachment to the wall of the egg capsule and onset of muscular crawling; Radula, onset of radula function. Arrow indicates sequence heterochrony between crawling and heart function in *P. acuta* (Smirthwaite et al., 2007).

*Lymnaea stagnalis* and *P. acuta* both pass through comparable physiological windows, separated by developmental events including ciliary driven rotation, cardiovascular function and muscular crawling. In *L. stagnalis*, there was no significant effect of temperature on energy within any frequencies during both ciliary driven rotation and cardiovascular function. However, during muscular crawling (i.e. when the embryo had attached to the wall of the egg capsule and commenced muscular crawling), there were significant increases in energy at 25°C relative to 20°C in frequencies within the ranges of 0.6–0.9 and 1.8–2.1 Hz. Additionally, significant increases in energy were observed during the onset of radula function in embryos reared at 25°C for frequencies within the range of 1.8–2.1 Hz (multivariate Kruskal–Wallis, *P*<0.00083; Fig. 2; Table S3).

In *R. balthica* embryos, we observed significant increases in energy at 25°C relative to 20°C within a broad range of frequency bands throughout development. Embryos during ciliary driven rotation also had greater energy at 25°C within the ranges of 0.1–1.7, 4.1–4.7 and 5.9–6.0 Hz. During cardiovascular function, there was a significant increase in energy at 25°C in frequencies within the ranges of 0.4–1.8 and 2.6–6.0 Hz. During muscular crawling, energy in frequencies within the ranges of 0.1–2.7 and 3.2–6.0 Hz were significantly increased at 25°C. Finally, following the onset of radula function embryos showed a significant increase in energy at 25°C in frequencies within the ranges of 0.3–1.1, 1.8–2.5 and 3.4–6.0 Hz (multivariate Kruskal–Wallis, *P*<0.00083; Fig. 2; Table S3).

Finally, in *P. acuta* during ciliary rotation, embryos exhibited a significant increase in energy at 25°C relative to 20°C in frequencies within the ranges of 0.1–0.4 and 4.4–6.0 Hz. During muscular crawling, temperature effects were limited to significant increases in energy at 0.5 and 4.6–5.0 Hz. However, after the appearance of cardiovascular function there were significant increases in energy at 25°C within frequencies within the ranges of 0.4–1.0 and 1.8–2.5 Hz. During radula function, energy was significantly greater at 25°C than 20°C in the frequencies of 0.5 and 2.0–2.7 Hz (multivariate Kruskal–Wallis, *P*<0.00083; Fig. 2; Table S3).

### Combinatorial analysis of EPTs

**Fig. 3.**
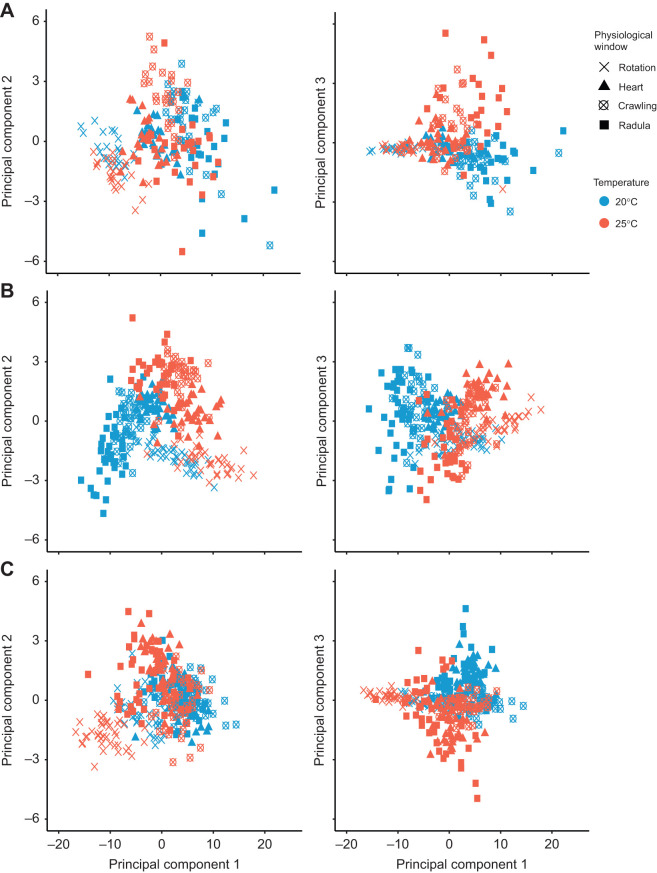
**Principal component analysis (PCA) of energy proxy trait data across species and physiological windows in development.** PCA was applied to mean energy within 60 temporal frequency bins at two temperatures (20°C and 25°C) and four physiological windows in development (ciliary driven rotation, crawling on the wall of the capsule, cardiovascular function and radula function) during the embryonic development of (A) *L. stagnalis*, (B) *R. balthica* and (C) *P. acuta*.

Multivariate analysis of EPT spectra were used to test for high-dimensional thermal and species-specific differences during multiple windows of physiological development. Reduction of EPT data (0–6.0 Hz) at each physiological window in development to three dimensions using PCA, revealed distinct clustering based on temperature and developmental stage. The first three principle components (PCs) of PCA analyses on each species cumulatively explained 93.15, 87.30 and 90.50% of the variance for *L. stagnalis*, *R. balthica* and *P. acuta*, respectively. For *L. stagnalis*, separation of points between temperatures during ciliary-driven rotation and muscular crawling was predominantly along the axis of PC2 (Fig. 3A). Variance along the axis of PC2 was driven by frequencies ranging from 0.03 to 1.0 Hz, indicating that temperature differences during these physiological windows of development were driven by changes in energy within these frequencies (Table S4). Furthermore, during radula function, differences between embryos reared at 20 and 25°C were driven by changes in energy within frequencies ranging from 1.8 to 2.1 Hz, given that points were separated predominantly along the axis of PC3, and variance along this axis driven by these frequencies (Table S4; Fig. 3A).

In *R. balthica*, during ciliary-driven rotation and cardiovascular function, embryos at different temperatures were principally separated along the axis of PC1, and these differences were driven predominantly by frequencies in the range of 1.8–2.5 Hz. During muscular crawling, separation of embryos at each temperature was distributed evenly across the axes of both PC1 and PC2, differences that were driven by changes in energy within frequencies ranging from 0.03 to 0.8 Hz (PC2) and 1.9 to 2.5 Hz (PC1). Additionally, during radula function, embryos reared at different temperatures were separated along the axis of PC3, variance in which was driven by changes in energy within frequencies of 0.1–0.6, 1.5–1.7 and 3.0–3.2 Hz (Table S4; Fig. 3B).

Finally, in *P. acuta*, during ciliary-driven rotation, frequencies ranging from 2.0 to 4.0 Hz were predominantly driving differences between embryos at 20°C and 25°C, given that these embryos were mainly separated along the axis of PC1. During cardiovascular function and radula function, embryos were separated along the axes of both PC1 and PC2, variance in which was driven by changes in energy within frequencies ranging of 2.0–4.0 and 0.03–1.0 Hz, respectively. Additionally, embryos during cardiovascular and radula function were driven by frequencies ranging from 2.0 to 2.4 Hz, separated along the axis of PC3 (Fig. 3C; Table S4).

## DISCUSSION

We applied a phenomics approach to test thermal sensitivity of the phenome of developing embryos of three species of freshwater snail with pre-established evolutionary differences in physiological event timings. Additionally, we aimed to understand how thermal responses of the phenome varied between physiological windows in development that vary in their observable phenotypes. EPTs revealed interspecific differences in relative sensitivities to chronic elevated temperatures, and differences in thermal responses between physiological windows in development. Additionally, temperature-induced changes to the timings of physiological development were identified from EPT time series. In summary, EPTs exhibited marked differences in the magnitude and direction of thermal effect between species and physiological windows in development.

### EPTs reveal interspecific differences in the developmental response to chronic elevated temperatures

Major interspecific differences were evident in the thermal sensitivity of time series of total energy. Total energy is the sum of energy across all frequencies for each hourly 30 s video, and it integrates all sources of biological movement present in video. Given that temperature affects rates of processes at every level of biological organisation, responses in total energy under chronically elevated temperatures is indicative of such broad-scale thermodynamic changes. *Radix balthica* showed the greatest magnitude of response in total energy from 20°C to 25°C compared with *L. stagnalis* and *P. acuta* (Fig. 1), suggesting heightened thermal sensitivity in embryos of *R. balthica* (Hochachka and Somero, 2002; Iverson et al., 2020). Lower magnitudes of change were observed in total energy in *P. acuta* and there were even reductions in total energy in *L. stagnalis* (Fig. 1A,C,D). The comparatively lower thermal sensitivity in total energy in embryos of *L. stagnalis* and *P. acuta* may reflect a number of scenarios. Firstly, this could represent considerably lower thermal sensitivity of rates of observable embryonic physiology and behaviour at these stages of development; however, this is unlikely given the obvious effects on energy at frequencies associated with cardiovascular function (Figs 2, 3; see Results). Secondly, this may indicate a decoupling of levels of overall embryo activity and maintenance of basic physiological function under chronically elevated temperatures (Pörtner, 2010). Rates of many observable organismal physiologies and behaviours continue to increase until a thermal optimum is reached, beyond which rates rapidly decline (Huey and Kingsolver, 1989; Angilletta, 2006). In species that can behaviourally thermoregulate or that experience relatively mild variations in temperature in their environments, behavioural thermal ranges are generally assumed to match physiological tolerance limits (Hernández and Bückle, 2002; Monaco et al., 2017). However, when behavioural thermoregulation is not an option, rates of activity may decline at lower temperatures than those of physiological function. For example, Monaco et al. (2017) showed that in six species of intertidal gastropod, the CT_max_ of crawling speed was less than that of heart rate. Furthermore, species occupying greater shore heights and therefore greater temperature extremes generally exhibited a greater degree of decoupling between these traits. Finally, the comparatively lower magnitude of change in total energy in embryos of *L. stagnalis* and *P. acuta* may indicate that embryos have already moved past their thermal optimum such that total energy appears to have declined (Angilletta, 2006). The observed reduction in total energy in embryos of *L. stagnalis* at 25°C may suggest some form of limitation on these embryos, thereby reducing energy allocated to gross rates of physiology and behaviour. Previous research applying EPTs to the embryonic development of *R. balthica* showed that development at 30°C resulted in a major reduction in energy across the whole period of embryonic development, indicating depressed rates of organismal movement, despite an increase in heart rate (Tills et al., 2018). This highlights a limitation of the methodology used in this study. Given that two temperatures were used (20°C and 25°C), we were unable to identify specific temperatures at which physiological performance began to decline, e.g. through the construction of a thermal performance curve (Angilletta, 2006). Consequently, future research could be directed towards establishing thermal performance curves for EPTs for these species. Given that frequencies within energy spectra correspond with different observable physiologies and behaviours, EPTs may provide an effective means with which to construct thermal performance curves for whole-organismal physiology and behaviour.

Our understanding of climate drivers on aquatic animals is based predominantly on studies of sexually mature adults, often ignoring earlier developmental stages. This is despite periods of early development showing equivalent, if not greater, sensitivities to numerous types of environmental change (Burggren, 2018, 2021). However, current approaches to phenotyping periods of early development are often not transferable between species that vary in their relative timings of development as they (a) fail to integrate the considerable structural and functional changes associated with embryonic development, and (b) use reductionist approaches centring on small numbers of observable phenotypes rather than integrating the widespread changes to observable phenotypes typically associated with the response to chronic elevated temperatures (Burggren, 1987; Spicer and Burggren, 2003; Forsman, 2015). In the present study, phenomics through the application of EPTs revealed major differences in the relative sensitivities of embryos of *R. balthica*, *L. stagnalis* and *P. acuta* to chronic elevated temperatures, as well as differences in thermal sensitivity between various physiological windows in these embryos. Whilst high-dimensional phenotyping approaches are well established for early developmental stages of model species such as the zebrafish *Danio rerio* (Xu et al., 2010; Peravali et al., 2011; Spomer et al., 2012), the nematode *Caenorhabditis elegans* (White et al., 2010; Olmedo et al., 2015) and the fruit fly *Drosophila melanogaster* (Chung et al., 2010; Levario et al., 2016), approaches that are transferable between non-model species of interest are lacking. The indiscriminate nature by which embryonic movements comprising observable physiologies and behaviours are captured by EPTs may facilitate transferability between non-model species of interest. Expansion of animal models beyond common model species, to support biological research across a greater breadth of diversity, would improve the evidence base for the effects of climatic change on early development (*sensu* Krogh, 1929; Burggren, 2021). However, there are still limitations associated with applying this approach to novel species. Although EPTs enable transferability between species and physiological windows of development, interpretation of their responses in a *de novo* sense does present difficulties. In the present study, the use of known physiological windows in development allowed for the interpretation of results within the context of a number of known embryonic physiologies and behaviours. However, if applied to a new species for which the developmental itinerary is not known, interpretation of the results in the absence of this ‘scaffold’ of developmental event timings becomes more difficult. Despite this, the capacity to integrate all observable forms of embryonic movement and analyse these in a combinatorial fashion has allowed for the detection of stress responses to various environmental toxicants (Rudin-Bitterli et al., 2014), and EPTs can be used in the detection of known aspects of organismal physiology (Ibbini et al., 2022).

Shifts in the relative timings of physiological development between embryos reared at 20°C and 25°C were also apparent from total energy time series. In *L. stagnalis*, increases in total energy associated with the onset of ciliary driven rotation, as well as decreases in energy associated with the onset of intermittent resting behaviours (J.C.S.M., unpublished observations), both commenced earlier in relative developmental time, reflecting an acceleration of the timings of these events at 25°C (Fig. 1). We also observed a decoupling of these two events in *R. balthica*, where the onset of ciliary-driven rotation remained unchanged, and the onset of intermittent resting accelerated at 25°C. Manual quantification of the timing of these events confirmed that differences mirrored these transitions evident in total energy time series (Table S2). Acceleration of the relative timings of developmental events has been observed in a number of species, and so has uncoupling of the timings of different developmental events. For example, in embryos of the herring *Clupea harengus* exposed to elevated temperatures, the timings of various developmental events exhibited different thermal sensitivities. Increased developmental temperature resulted in differences to the relative timing of organogenesis (spinal cord, pectoral fin buds and myotomal muscle fibres), whilst the timings of tissue differentiation remained almost unchanged (Johnston, 1993). EPT spectral time series indicate that although for *L. stagnalis*, growth at a higher temperature results in an acceleration of the majority of the developmental itinerary, for *R. balthica* (and *P. acuta*) there is a decoupling of these major developmental transitions in relative developmental time. This is of particular interest as plasticity in the timings of development may act as a driver of evolutionary change (Spicer and Rundle, 2007; Spicer et al., 2018). Selection typically acts on multiple traits simultaneously (Lande and Arnold, 1983; Phillips and Arnold, 1999), and given that EPTs integrate a number of observable embryonic physiologies and behaviours, it is not unreasonable to question whether EPTs, and temperature-induced changes in the timings of total energy, may act as objects of multivariate selection.

### Differences in thermal responses between physiological windows in development correspond with ontogenetic changes to observable phenotype

Changes in observable embryonic phenotype as development progressed were reflected in differences of the response of EPTs between physiological windows in development. For example, during muscular crawling in *L. stagnalis*, temperature differences were predominantly driven by changes in energy within frequencies likely corresponding with observable physiologies including body flexing and mantle muscle control (0.6–0.9 Hz) (Meshcheryakov, 1990; Smirthwaite et al., 2007). Conversely, during radula function, temperature differences were mainly driven by changes in energy at 1.8–2.1 Hz, frequencies corresponding with observable heart beating (Voronezhskaya et al., 2007). Similarly, in *P. acuta*, changes in energy following the onset of cardiovascular and radula function were also mainly within frequencies associated with a heartbeat (1.8–2.5 Hz and 2.0–2.7 Hz, respectively) (Seeland et al., 2013), as well as changes in energy within frequencies likely associated with body flexing and mantle muscle control (0.4–1.0 Hz). Rather than targeting specific aspects of embryonic physiology or behaviour, spectral phenotyping through the application of EPTs quantifies changes in pixel value fluctuations, thereby facilitating transferability between stages of development, despite considerable differences in observable phenotype.

EPTs enabled the continuous measurement of phenotypic change across major transitions in the observable phenotype. However, comparison of EPT spectra during discrete physiological windows also enabled robust analysis of EPT spectra during periods consisting of specific observable embryonic phenotypes. Embryonic development encompasses unrivalled levels of structural and functional change, rendering the continuous quantification of environmental effects on developmental phenotype particularly problematic (Burggren, 1987; Spicer and Burggren, 2003). Assessment of phenotypic responses throughout embryonic development often necessitates quantification of changes in specific traits or broad-scale indicators of organismal performance, for example rates of oxygen consumption (Pörtner et al., 2011) and tolerance limits to forms of environmental stress (Kuramoto, 1978; Hammond and Hofmann, 2010; Storch et al., 2011; Truebano et al., 2018), at discrete times or stages in developmental time. In his recent review, Burggren (2021) highlighted that a significant limitation of physiological measurements at discrete points in development is that they may be inaccurate, and that by considering development as a continuum, physiological measurements can be put into the context of an organism's entire development. Continuous quantification of phenotypic change rather than measuring discrete points in developmental time will be central to robust measures of developmental responses to climatic change (Burggren, 2021).

Finally, the observed changes in EPTs under elevated temperatures stimulate the idea that such changes in EPT spectra may have implications for organismal performance and fitness. Previous relationships were established between EPTs and a developmental outcome (growth rate), suggesting that EPTs may provide a visual proxy for rates of biochemical energy turnover in developing embryos (Tills et al., 2021). Here, we observed differences in both the magnitude of change in total energy and shifts in EPT time series in relative developmental time following exposure to chronic elevated temperatures. Allocation of energetic reserves to various behavioural and physiological functions is hypothesised to be a careful trade-off based on the environmental conditions under which an organism finds itself (Brafield and Llewellyn, 1982). If EPTs are directly related to biochemical energy turnover in developing embryos, visual quantification of levels of biochemical energy turnover within different temporal frequencies could provide useful proxies for how energy is allocated into various processes throughout the whole period of development, rather than characterising energetic turnover at discrete points (Attard and Hudon, 1987; Stackley et al., 2011). Such a proxy may provide useful insights into the effects of climatic change on performance and fitness of developing embryos, via quantification of biochemical energetic turnover.

### Conclusions

Assessing phenotypic responses to elevated temperatures during early development should be central to predicting how species might respond to climatic change (Burggren, 2018). The application of EPTs revealed interspecific differences in relative sensitivities to chronic elevated temperatures, temperature-induced changes in the relative timings of development, and differences in thermal responses between physiological windows in development that each largely coincide with ontogenetic differences in observable phenotypes. Crucially, EPTs provided an approach to high-dimensional organismal phenotyping that is transferable between species that vary in their early development, and between physiological windows in development that vary in their observable phenotypes. Furthermore, the indiscriminate nature of EPTs results in the integration of all observable embryonic phenotypes, and analysis of these data in a combinatorial fashion, rather than focusing on small numbers of observable embryonic phenotypes. Understanding the broader implications of climate change on early life stages of aquatic animals requires phenotyping approaches that are applicable to non-model species favoured by the Krogh principle (Feder, 2006; Burggren, 2021), and to assess phenotypic change continuously through early development, rather than simplifying the dynamic process of embryonic development into small sets of discrete developmental stages.

## Supplementary Material

10.1242/jexbio.245612_sup1 Supplementary information
