# Supplementary material for: A phenomics approach reveals interspecific differences in integrated developmental responses to chronic elevated temperatures
Source: J Exp Biol. 2023 Jun 29;226(12):jeb245612. doi: 10.1242/jeb.245612 (PMC10323248; doi:10.1242/jeb.245612)
Supplement: Supplementary information [file jexbio-226-245612-s1.pdf]

Table S1. Summary of TukeyHSD results investigating the time point specific effects of temperature on total energy, for embryos of *Lymnaea stagnalis* (20°C: n = 32, 25°C: n = 26), *Radix balthica* (20°C: n = 40, 25°C: n = 37) and *Physella acuta* (20°C: n = 43, 25°C: n = 41).

Click here to download Table S1

Table S2. Raw data for the absolute and relative timings of physiological events used in this study. Absolute timings of developmental events were manually by observation of video of developing embryos of *Lymnaea stagnalis* (20˚C N = 32, 25˚C N = 26), *Radix balthica* (20˚C N = 40, 25˚C N = 37) and *Physella acuta* (20˚C N = 43, 25˚C N = 41). Absolute timings data were used to calculate relative timings, by standardising between the 4-cell stage and hatching.

Click here to download Table S2

Table S3. Summary of multivariate Kruskal-Wallis results investigating frequency specific effects of temperature on energy, at 4 key physiological windows in development (ciliary driven rotation, crawling on the wall of the capsule, cardiovascular function, radula function) for embryos of *Lymnaea stagnalis* (20°C: n = 32, 25°C: n = 26), *Radix balthica* (20°C: n = 40, 25°C: n = 37) and *Physella acuta* (20°C: n = 43, 25°C: n = 41).

Click here to download Table S3

Table S4. PCA loadings data frequency specific EPT data across species, temperature and physiological windows in development. PCA was applied to mean energy within 60 temporal frequency bins at 20 and 25˚C, and 4 physiological windows in development (ciliary driven rotation, crawling on the wall of the capsule, cardiovascular function, radula function), for embryos of *Lymnaea stagnalis* (20°C: n = 32, 25°C: n = 26), *Radix balthica* (20°C: n = 40, 25°C: n = 37) and *Physella acuta* (20°C: n = 43, 25°C: n = 41).

Click here to download Table S4

Journal of Experimental Biology • Supplementary information
